# Effect of skill proficiency on motor imagery ability between amateur dancers and non-dancers

**DOI:** 10.3389/fpsyg.2022.899724

**Published:** 2022-08-12

**Authors:** Xiaoling Mao, Shaoxu Huang, Mingkun Ouyang, Yangqiu Xie, Xinhua Tan

**Affiliations:** ^1^Education Center for Mental Health, Guangxi Minzu University, Nanning, China; ^2^School of Education Science, Guangxi Minzu University, Nanning, China; ^3^Guangxi Key Laboratory of Processing for Non-ferrous Metals and Featured Materials, Guangxi University, Nanning, China; ^4^School of Information Science and Engineering, Yanshan University, Qinhuangdao, China

**Keywords:** motor imagery, visual motor imagery, kinesthetic imagery, VMIQ, amateur dancers

## Abstract

Evidence has shown that athletes with high motor skill proficiency possess higher motor imagery ability than those with low motor skill proficiency. However, less is known whether this superiority in motor imagery ability emerges over amateur athletes. To address the issue, the present study aimed to investigate the individual differences in motor imagery ability between amateur dancers and non-dancers. Forty participants completed a novel dance movement reproduction task and measures of the vividness of visual imagery questionnaire (VVIQ) and the vividness of motor imagery questionnaire (VMIQ). The results showed that, relative to non-dancers, amateur dancers had higher ability of motor imagery to reproduce the lower-limb and upper-limb dance movements during the dance movement reproduction task. Besides, amateur dancers displayed higher abilities of the visual motor imagery and the kinesthetic imagery, but comparable visual imagery ability as the non-dancers. These findings suggest that the mental representation of motors but not the visual is affected by the motor skill levels, due to the motor imagery practice in sports amateurs.

## Introduction

Motor imagery refers to the mental rehearsal of movements in absence of overt muscular activation (Jeannerod, 1994). Motor imagery is divided into kinesthetic imagery (KIN; or termed “internal visual imagery”) and visual motor imagery (EVI; or termed “external visual imagery”; Lang, 1979; Hardy and Callow, 1999; Féry, 2003; Stevens, 2005; Roberts et al., 2008). KIN is defined as “a person actually imagines being inside his or her body and experiences those sensations which might be expected in the actual situation” (Mahoney and Avener, 1977, p. 137). As the name implies, KIN means that a person imagines conducting a task from the self-centered frame of reference, which is associated with a kinesthetic representation of movement. In contrast, EVI reflects viewing the movement in a viewer-centered frame of reference, which involves a visual motor representation of movement (Gaggioli et al., 2013).

### Mental practice with motor imagery

Motor imagery practice is defined as the mental practice of motor imagery contents aimed to improve motor performance. Considerable research has shown that motor imagery practice is a widely used psychological skills for enhancing performance and motor skills learning in a variety of sport settings, suggesting that the ability to represent internal representations of motor can improve following exposure to imagery practice (Bohan et al., 1999; Cumming and Hall, 2002; Mizuguchi et al., 2012; Gaggioli et al., 2013; Frank et al., 2014; Dana and Gozalzadeh, 2017). For example, 70–90% athletes report that regular motor imagery practice remarkably improves their motor performance (Mizuguchi et al., 2012). Moreover, evidence from non-athletes has established the usefulness of motor imagery practice in motor rehabilitation among healthy people (Macuga and Frey, 2012; Guillot et al., 2014) and patients with Parkinson’s disease (Helmich et al., 2007; Roosink and Zijdewind, 2010) and poststroke hemiparesis (Guttman et al., 2014), suggesting that the ability to generate mental imagery of movement can also be greatly improved even when actual motor function is impaired or lost.

The benefits of motor imagery practice in motor performance can be explained by the functional equivalence hypothesis (Johnson, 1982; Williams et al., 2011). According to the hypothesis, motor imagery, and motor execution are functional equivalent. They conform to fitts’ law (Fitts, 1954), and take the similar time to complete motor tasks (Decety and Michel, 1989). Behaviorally, the mental effort required during simulating movements is highly associated with the amount of force which is carried out in motor execution (Decety and Lindgren, 1991). Neurally, motor imagery and motor execution elicit similar brain networks of motor-related regions, such as the supplementary motor area (SMA), premotor cortex (PM), and cerebellum (Hanakawa et al., 2003; Lacourse et al., 2005; Guillot et al., 2009). To put it differently, although without any real movement or muscle activation, motor imagery induces analogous physiological responses and neural networks that share with those activated by actual motor execution, and then facilitates the acquisition or improvement of motor skills (Ruffino et al., 2017).

Empirical evidence has shown that motor imagery practice exerts a positive influence on dance performance (Girón et al., 2012; Abraham et al., 2016, 2017). Motor imagery practice offers dancers great potential benefits, such as improving mean strength (Fontani et al., 2007), dance skill accuracy (Yoo et al., 2001), increasing self-confidence (Guillot and Collet, 2008), and decreasing stress (Magill and Anderson, 2007). KIN and EVI are two independent components of the motor imagery (Roberts et al., 2008), and Girón et al. (2012) showed that the KIN and EVI contribute to different aspects of dance performance. Especially, KIN provided dancers with much proprioceptive feedback information, leading to the increased hip rotation for pliés, while EVI offered dancers much salient information, leading to the increased hip rotation for sautés. Given that KIN and EVI are two related but independent constructs of the motor imagery (Roberts et al., 2008), it remains to be demonstrated whether and how these two motor imagery abilities are affected in amateur dancers with differences in motor imagery practice or experience. Research on this domain is still insufficient. To address the gap, the present study aimed to extend existing research by examining the effect of motor imagery practice on motor imagery ability between amateur dancers and non-dancers.

### Motor imagery practice and imagery ability

A large body of research has empirically confirmed that imagery practice shapes the imagery ability or the capability for “forming vivid, controllable images and retaining them for sufficient time to affect the desire imagery rehearsal” (Hall et al., 1992; Isaac and Marks, 1994; Morris, 1997; Eton et al., 1998; Oishi and Maeshima, 2004; Yu et al., 2016). Studies have shown that frequent use of the imagery improve individuals’ ability to imagery vivid actions (Isaac and Marks, 1994; Stefanello et al., 2010). Isaac and Marks (1994), using vividness of visual imagery and movement imagery questionnaires, for example, provided evidence that individuals with higher motor skills possessed a better capacity of motor imagery. They found that elite athletes (e.g., gymnastics and trampoline) reported significantly higher vivid imagery than control groups. In addition, non-athletes, such as physical education students, also exhibited higher vivid movement imagery (i.e., KIN) than students from other disciplines (e.g., English and physics; Isaac and Marks, 1994). Similar results are obtained with approach of mental chronometry (Ozel et al., 2004; Malouin et al., 2008). For example, Ozel et al. (2004) showed that both open- and closed-skilled athletes performed significantly faster than non-athletes on discrimination and actual rotation, two subprocesses of the mental rotation task. The authors attributed these findings to the possibility that athletes have developed greater ability to perform mental imagery transformation (i.e., imagery controllability) due to the imagery practice. Arvinen-Brown et al. (2007) provided evidence that athletes and novice differ on imagery use regardless of whether participating in either open- or closed-skilled sports. Taken together, these findings above suggest that individuals with high motor skills possess a higher motor imagery ability, compared with those with low motor skills. One potential reason for the superiority in motor imagery is that individuals with high motor skill levels expose to much motor imagery practice in daily life, due to the cognitive and physical demands of movement (Gregg and Hall, 2006; Yu et al., 2016).

To our knowledge, it is unclear whether amateur dancer with much motor imagery practice shows high ability of motor imageries, such as KIN and EVI, compared with non-dancers. Previous studies have shown that the benefits of KIN in closed motor skills learning (e.g., gymnastics and figure skating), and EVI in open motor skill learning (e.g., football, basketball, and squash; Spittle, 2001; Coelho et al., 2007; Dana and Gozalzadeh, 2017). In addition, studies showed that KIN was more effective than EVI for the rapidly changed movements (Callow et al., 2013) and the precise spatial locations of movements (Hardy and Callow, 1999). Given that dance movement such as ballet and hip-hop relies heavily on closed skills, using KIN, rather than EVI, should be more effective for the acquisition and performance of dance. One study found that the learning of aerobics based on EVI was more effective for the novice, yet, as they practice more, the training based on KIN was better (Zhang, 2015). The reason is that once the amateur dancers are familiar with the motor skills, the KIN will gradually take the advantage to gain a more accurate and sophisticated motor (Hardy and Callow, 1999). Hence, following this logic, we hypothesized that the ability to utilize KIN would be higher for amateur dancers than non-dancers. While for the EVI, though it could provide additional visuospatial information which might enable individuals to “see” the precise spatial positions and movements of the dance, it is not always effective for dancer to mentally rehearse the relative complex movements of dance. Therefore, it is reasonable to postulate that the ability to use EVI to improve dance performance may be same for amateur dancer and non-dancers.

### The present study

In the previous studies, motor imagery ability was measured mainly using subjective self-report questionnaires. Although subjective self-report questionnaires, such as vividness of visual imagery questionnaire (VVIQ; Isaac et al., 1986) and movement imagery questionnaire (VMIQ; Hall and Martin, 1997), are widely used for measuring individual differences in motor imagery, the validity of these subjective measures have criticized by theorists, who reported that subjective measures of motor imagery have little to do with spatial test performance (Dean and Morris, 2003). Besides, self-report measures of motor imagery are sensitive to individual differences in verbal comprehension. Given the weaknesses of subjective self-report measures, researchers proposed the combination of subjective and objective measures for dance research on motor imagery (Carey et al., 2019). Regarding objective measure of motor imagery, previous studies measured the individual differences in motor imagery ability based on the accuracy of actual movement, by using the scores on various mental chronometry tasks such as ballet (Lemos et al., 2017) and stepping movements (Malouin et al., 2008). However, the results of these studies are likely to mirror different aspects of motor imagery, other than vividness or accurate imagined movement, since participants can generate the start and the end of motor imagery even when their movements are not accurate. In the present study, a novel dance movement reproduction task as the objective measure was used to assess the individual differences in motor imagery ability between amateur dancers and non-dancers. In this task, participants were asked to learn novel dance movements that were presented by cartoons, and then to reproduce dance movements as correctly as possible. Motor imagery ability was measured by evaluating the number of actual correct movements based on the motor representation that was obtained by referencing the working memory for dance movement. The dance movement reproduction task is valid, since the procedure of the task is exactly the same as the one used by Cortese and Rossi-Arnaud (2010). Previous studies using the similar dance movement reproduction task reported that kinesthetic and visual motor imageries were likely to be used by participants (Mizuguchi et al., 2016; Orlandi et al., 2020a). According to the model of motor skill learning proposed by Hikosaka and colleagues, specific aspects of motor imagery such as visual and kinesthetic motor imageries are reported to be used to improve actual performance of dance movements (Hikosaka et al., 1999, 2003). Participants may use kinesthetic motor imagery to mentally rehearse the muscle contractions and stretching, and use visual motor imagery to visualize the dance movements (Guillot et al., 2009). As studies showed the validity of the dance movement reproduction task in measuring the motor imagery ability (Mizuguchi et al., 2019, 2021), we hypothesized that the motor imagery ability measured by the dance movement reproduction task would differ in terms of the dance-related movement experiences (i.e., motor imagery practice). More precisely, if the abilities to generate mental motor imagery was significantly higher in amateur dancers than in non-dancers, the actual performance of the dance movement reproduction task would be better in amateur dancers than non-dancers.

We chose amateur dancers as the sample population for the following reasons: (1) dancers were reported to use imagery more than athletes (Cumming and Williams, 2013); (2) dancers of all ages and skill levels use motor imagery practice for improving the performance of dance movements in their daily practices (Pavlik and Nordin-Bates, 2016); (3) skill levels of dancers are associated with the frequency of the motor imagery use. For example, more proficient dancers reported using motor imagery more frequently than their less proficient counterparts, leading to a higher ability of motor imagery (Nordin and Cumming, 2006; Paris-Alemany et al., 2019a; Orlandi et al., 2020a); and (4) to the best of our knowledge, no previous studies have investigated the individual differences in motor imagery between amateur dancers and non-dancers who vary in dance skill levels and experience.

Taken together, an objective measure (i.e., a dance movement reproduction task) in combination of subjective measures (i.e., VVIQ and VMIQ) was used in the present study to assess the individual differences in motor imagery ability between amateur dancers and non-dancers. We aimed to determine whether and how motor imagery ability could be influenced in individuals with differences in dance-related movement experience (i.e., motor imagery practice).

## Materials and methods

### Participants

In the present study, we used a convenient sampling method to recruit forty college students from one university in Guangxi province, China. There were 18 amateur dancers (i.e., hip-hop dance students; 8 men and 10 women, aged 18–22 years, mean age 19.67 years, SD = 2.03). All participants were elementary level dancers and had completed a dancing program of 1.5 years on average with 6.2 h per week of training. There were another 22 aged matched control participants (10 men and 12 women, aged 19–23 years, mean age 20.1 years, SD = 2.18), who had no dance training experience. All participants were right-handed and had normal or corrected-to-normal vision. This study was approved by the Research Ethics Committee of the Guangxi university for nationality, and all the participants signed an informed consent form.

### Measures

#### The vividness of visual imagery questionnaire

The vividness of visual imagery questionnaire developed by Marks (1973) was used to measure participants’ visual imagery. It has shown a satisfactory reliability and validity among Chinese adolescents (Song and Zhang, 2002; He et al., 2012). The VVIQ consists of 16 items which are clustered in four groups in which participants visualize and rates the vividness of the image formed in the mind when thinking about specific scenes and situations (e.g., figures and sky). Each item was rated on a *5*-point Likert scale ranging from *1* (*perfectly clear and vivid as normal vision*) to *5* (*no image at all, you only know that you are thinking of the skill*). Each item was imaged and rated once with open and once with closed eyes. Responses across the 16 items were summed, with lower score indicating a higher visual imagery. In the present study, the internal consistency reliability for the scale was 0.89, the test reliability was 0.76, and the content validity was 0.77 (McKelvie, 1995).

#### The vividness of movement imagery questionnaire

The vividness of movement imagery questionnaire (VMIQ) compiled by Isaac et al. (1986) was used to assess movement imagery ability. The questionnaire contains 24 items which are divided into six groups, with four items in each group. The groups range from items relating to basic body movements (e.g., jumping) to items relating to demanding control in aerial situations (e.g., jumping off a high wall). Participants were required to image each item twice: first by imagining watching somebody else perform the movement (i.e., *visual motor imagery*) and second by imagining performing the movement themselves (i.e., *kinesthetic imagery*). Each item is rated on a 5-point Likert scale ranging from *1* (*perfectly clear image and vivid as normal vision*) to *5* (*no image at all, you only know that you are thinking of the skill*), with lower summed scores indexing higher vividness of movement imagery. The VMIQ has shown acceptable reliability and validity. The test–retest reliability of VMIQ was 0.76 and the concurrent validity with VVIQ was 0.81 (Isaac et al., 1986).

#### The dance movement reproduction task

In the dance movement reproduction task, participants were required to generate the mental representations of dance movements and then reproduce (or retrieve) the dance movements based on the movements on picture in the dance movement learning phase. Ten cartoons in form of GIF were used in this study as experimental materials to control the individual differences in familiarity with dance movements (Paris-Alemany et al., 2019b). Each cartoon depicts both upper and lower body movements (or gestures) and no body movement repeated twice. The individual differences in familiarities with dance movements were examined on a 5-point Likert scale from 1 (*unfamiliar*) to 5 (*highly familiar*), and no statistically significant difference was observed, suggesting that amateur dancers and non-dancers were similarly familiar with the dance movements (*t* < 1, *p* > 0.05). Besides, the difficulty of dance movements on cartoons was rated by three professional dancers who had a dance experience of at least 8 years, with the results showing that the difficulty of dance movements was classified as easy, since the movements to learn requires low cognitive demands of attention.

### Procedure

Firstly, participants filled measurements of VVIQ and VMIQ. After completing the measurements, a dance movement reproduction task was conducted by participants. In the task, each cartoon was presented for 5 s, then a white fixation cross (+) was presented during which participants were asked to form mental representation of dance movements on picture and then reproduce them as correctly as possible. The time interval between cartoons ranged from 20 s to 30 s to prevent interference from dance movements in spatial working memory (Smyth and Pendleton, 1994). The whole process of the task was video-taped (Gaggioli et al., 2013). Participants’ dance performance was evaluated by three professional dancers in reference to the actual dance movements on cartoons. If a dance movement was correctly reproduced by participants, participants scored one point, otherwise, they scored zero point. The experiment was conducted single blind in that the raters did not know which participants were dancers or non-dancers (Lemos et al., 2017). The inter-rater reliability among three raters was calculated, and results showed that the reliability was acceptable with Cronbach’s alpha coefficient ranging from 0.87 to 0.93.

## Results

### The results of the VVIQ

**Figure 1:**
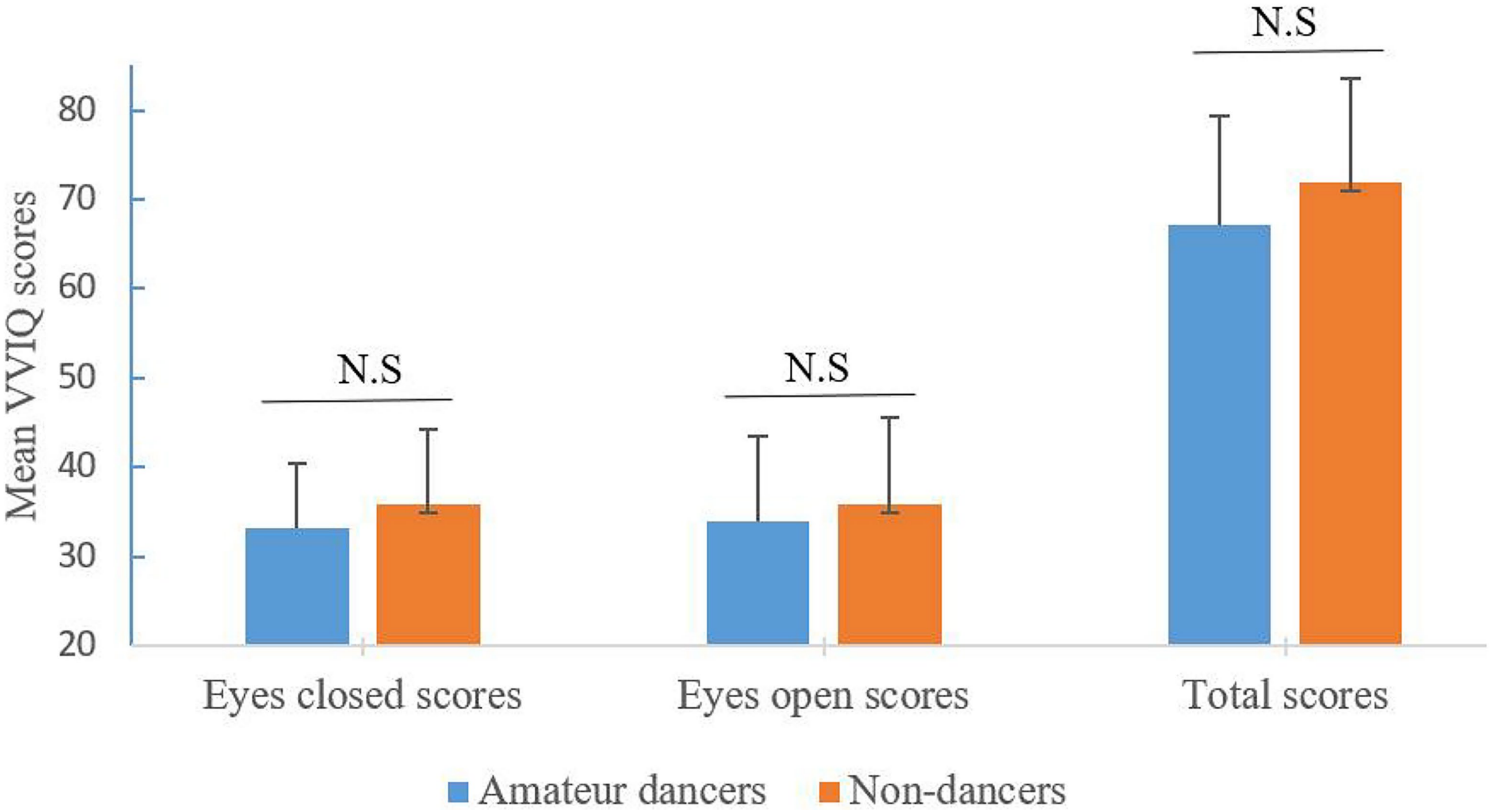
Individual differences in mean VVIQ scores between amateur dances and non-dancers. N.S indicates no significant difference, *p* > 0.05.

We analyzed the participants’ vividness of the VI, the results were presented in Table 1 and Figure 1. A 2 group (Amateur dancers vs. Non-dancers) **×** 2 imagery condition (eyes closed vs. eyes open) analysis of repeated measures ANCOVAs (with gender as a covariate) for the VVIQ scores showed that neither the main effect for group [*F*(1, 37) = 1.868, *p* = 0.18, *η*^2^ = 0.048] nor the main effect for imagery condition (*F* < 1) reached statistical significance. Similarly, the interaction effect between group and imagery condition did not reach statistically significant difference (*F* < 1).

**Table 1:** Mean VVIQ scores for eyes closed and open conditions (M ± *SD*).

Imagery conditions		Eyes closed scores	Eyes open scores	Total scores
Group	Amateur dancers	33.28 ± 7.08	33.94 ± 9.53	67.22 ± 12.12
	Non-dancers	35.94 ± 8.30	35.85 ± 9.83	71.95 ± 11.61

Due to the problems with null hypothesis significance testing (NHST), it is difficult for researchers to draw inferences from null findings. Bayesian analysis offers a clear estimate of the degree of confidence that the data support the null hypothesis on the condition that NHST fails to reject this hypothesis (Masson, 2011). Bayesian analysis can directly evaluate the relative strength of evidence favoring either the null or alternative hypothesis. The value of the Bayes factor greater than 3 (i.e., BF_01_ > 3 or BF_10_ < 1/3) is considered as “some evidence” supporting the null findings (Jeffreys, 1961). For example, the value of the Bayes factor is equivalent to 3 indicating that the data are 3 times more likely under null hypothesis than under alterative hypothesis. In the present study, the Bayesian analysis was conducted by computing ANCOVAs for the factors of group and imagery condition, and their interaction term. The results showed that for the main effects of group and imagery condition, the Bayes factor is 1.833 and 4.108, respectively, and for the interaction term, the Bayes factor is 1.547. Thus, there is some evidence favoring the null hypothesis over the alternative hypothesis. These results above indicated that in some ways the visual imagery for both dancers and non-dancers is virtually identical, both in the eyes closed and eyes open conditions.

With respect to the total scores of VVIQ, comparisons between two groups were tested by one-way ANCOVA analysis with gender as a covariate. The result revealed that the ability of VI was approximately the same for both amateur dancers and non-dancers [*F*(1, 37) = 1.868, *p* = 0.18, *η*^2^ = 0.048]. This outcome was further supported by the Bayesian analysis, showing that the Bayes factor of group was 1.701. In the following statistical analysis, Bayesian analysis was also applied.

### The results of the VMIQ

**Figure 2:**
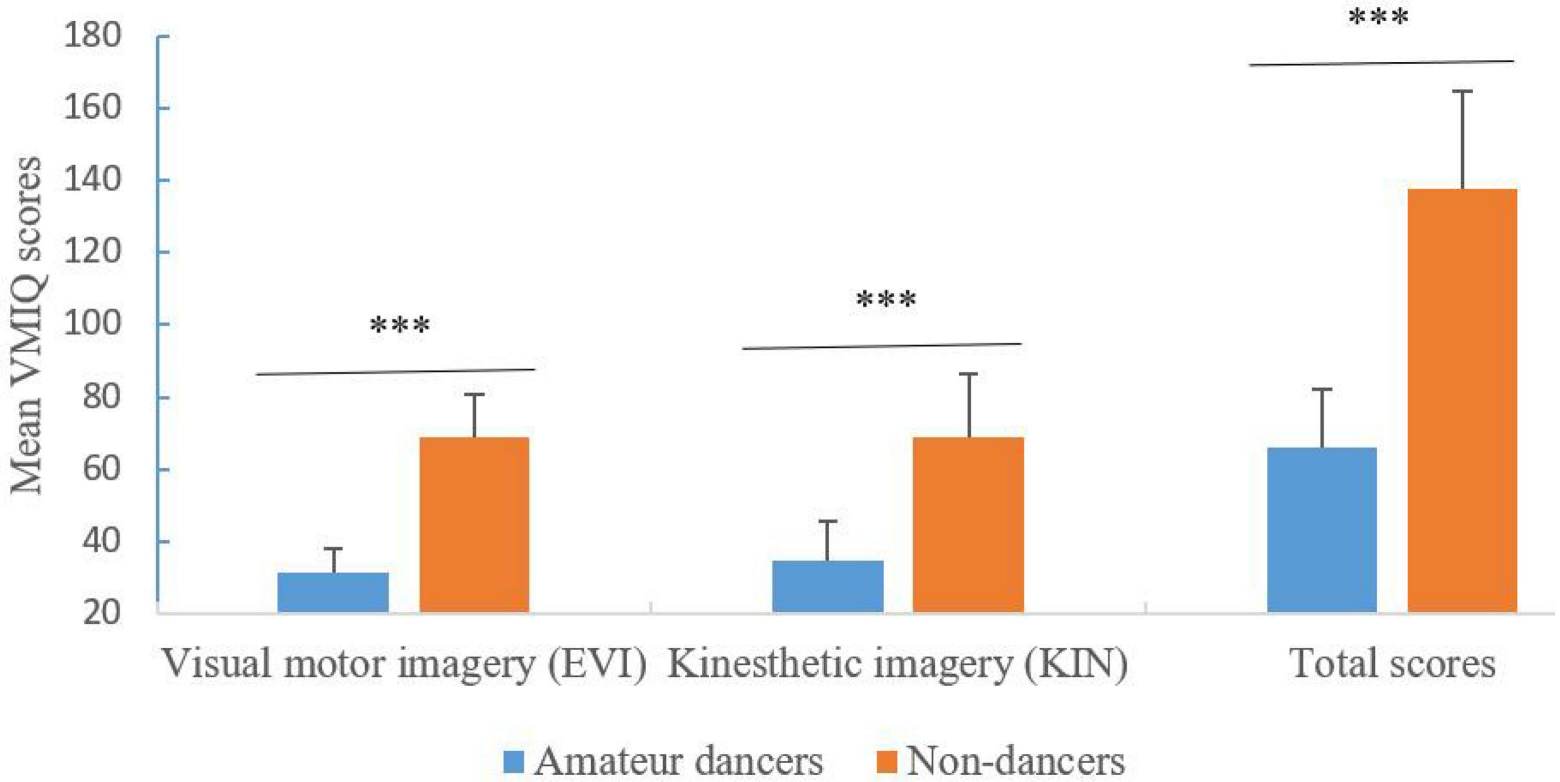
Individual differences in mean vividness of motor imagery questionnaire (VMIQ) scores between amateur dances and non-dancers. Asterisks indicate significance of the difference, ^***^p < 0.001.

We analyzed the vividness of motor imagery, the results were presented in Table 2 and Figure 2. A 2 group (Amateur dancers vs. Non-dancers) **×** 2 type of imagery (*Visual motor imagery* vs. *Kinesthetic imagery*) analysis of repeated measures ANCOVAs (with gender as a covariate) for the VMIQ scores showed that the main effect for group was significant [*F*(1, 37) = 98.587, *p* < 0.001, *η*^2^ = 0.727; Bayes factor BF_10_ = 2.47 × 10^9^]. The imagery scores were lower in amateur dancers compared with non-dancers. However, neither the main effect for type of imagery (*F* < 1; Bayes factor BF_10_ = 0.294) nor the interaction effect between group and type of imagery did reach statistically significant difference (*F* < 1; Bayes factor BF_10_ = 0.231). The results suggest that, irrespective of the type of imagery, the motor imagery performance was better in the dancers compared to that in the non-dancers.

**Table 2:** Mean VMIQ scores (*M* ± *SD*).

Types of imagery		Visual motor imagery (EVI)	Kinesthetic imagery (KIN)	Total scores
Group	Amateur dancers	31.50 ± 6.68	34.66 ± 11.15	66.17 ± 15.94
	Non-dancers	69.00 ± 11.92	68.86 ± 17.54	137.86 ± 26.73

With respect to the total scores of VMIQ, comparisons between two groups were tested by one-way ANCOVA analysis with gender as a covariate. The result showed that the ability of motor imagery was higher for amateur dancers than non-dancers [*F*(1, 37) = 98.59, *p <* 0.001, *η*^2^ = 0.727, Bayes factor BF_10_ = 1.50 × 10^9^].

### The results of the dance movement reproduction task

**Figure 3:**
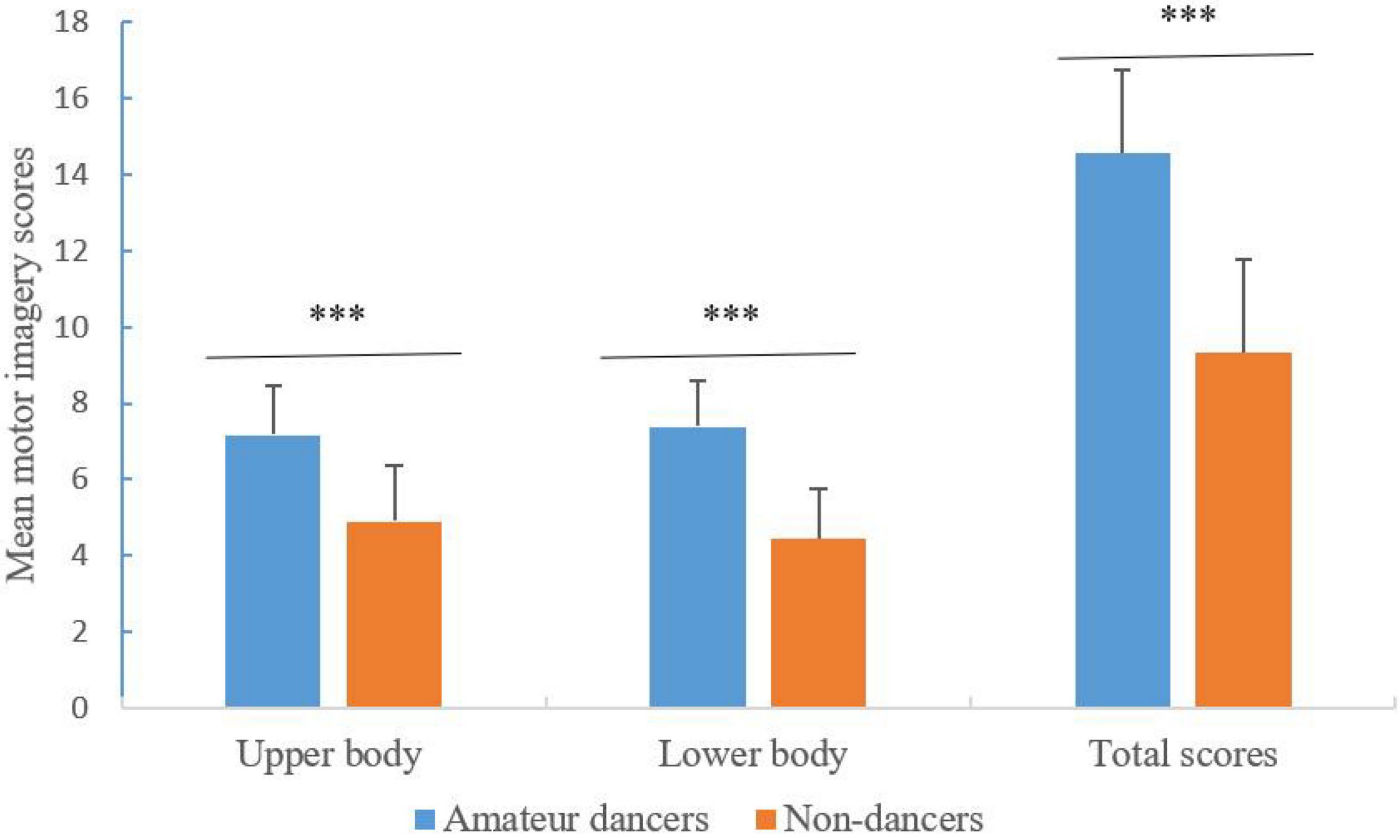
Individual differences in motor imagery between amateur dances and non-dancers assessed by the dance movement reproduction task. Asterisks indicate significance of the difference, ^***^p < 0.001.

We then analyzed the participant’ motor imagery based on the actual dance movements reproduced, the results were presented in Table 3 and Figure 3. A 2 group (Amateur dancers vs. Non-dancers) **×** 2 body position (upper body vs. lower body) analysis of repeated measures ANCOVA (with gender as a covariate) showed that the main effect for group was significant [*F*(1, 37) = 66.647, *p* < 0.001, *η*^2^ = 0.643; Bayes factor BF_10_ = 76133.47], indicating the ability of motor imagery was higher for amateur dancers than non-dancers. However, both the main effect for body position (*F* < 1; Bayes factor BF_10_ = 0.635) and the interaction effect between group and body position [*F*(1, 37) = 3.769, *p* = 0.06, *η*^2^ = 0.092; Bayes factor BF_10_ = 1.777] did not reach statistically significant. With respect to the total scores of motor imagery, comparisons between two groups were tested by one-way ANCOVA analysis with gender as a covariate, again showing that the ability of motor imagery is higher for amateur dancers than for non-dancers [*F*(1, 37) = 66.83, *p* < 0.001, *η*^2^ = 0.569; Bayes factor BF_10_ = 246938.37].

**Table 3:** Mean motor imagery scores for upper and lower body movements (*M* ± *SD*).

Body positions		Upper body	Lower body	Total scores
Group	Amateur dancers	7.17 ± 1.30	7.39 ± 1.20	14.56 ± 2.20
	Non-dancers	4.90 ± 1.45	4.45 ± 1.32	9.35 ± 2.43

To illustrate the specific components of imagery underlying dance movement reproduction, we examined the correlations between total scores on dance movement reproduction task and imagery scores for VVIQ and VMIQ, respectively. To be specific, there was no significant correlation between total scores on dance movement reproduction task and imagery scores for VVIQ [*r*(40) = −0.237, *p* = 0.141], suggesting that visual imagery might play little role in reproducing dance movements. However, the results found significantly negative correlations between total scores on dance movement reproduction task and imagery scores for VMIQ, with the correlations between performance of dance movement and visual motor imagery [*r*(40) = −0.342, *p* = 0.031] and between performance of dance movement and kinesthetic motor imagery [*r*(40) = −0.381, *p* = 0.015] being −0.342 and − 0.381, respectively. These results suggest that visual and kinesthetic components of motor imagery may be used by participants during dance movement reproduction.

## Discussion

The present study examined whether and how the motor imagery ability is affected by the individual differences in dance skill levels, using the combined approaches of self-report measures and a dance movement reproduction task. The results showed that amateur dancers outperformed non-dancers at the number of correctly reproduced dance movements. Besides, amateur dancers displayed higher abilities of visual motor and kinesthetic motor imageries, but comparable ability of visual imagery over non-amateurs. The study provides evidence for the higher levels of motor imagery ability in individuals with high levels of motor skill, due to much motor imagery experience in exercise and sports.

### The individual differences between amateur dancers and non-dancers in visual imagery ability

The results showed that the visual imagery scores were equivalent to amateur dancers and non-dancers, suggesting that participants are equally able to use visual imagery, and the visual imagery may not be affected by dance skill levels. The possible reason for this null effect is that the sport skill in the present study is dance, which is treated as a closed skill sport characterized by repetitive movements in a relatively unchanging environment (Nuri et al., 2013). Compared with other imageries such as motor imagery, visual imagery practice has been shown to be less effective in closed motor skill and sport performance (Coelho et al., 2007; Yu et al., 2016). Thus, amateur dancers may expose less to visual imagery during mental practice in dance movement learning, leading to a similar result as the non-dancers regarding visual imagery scores. The result is consistent with previous studies using the same measures conducted by Eton et al. (1998). Especially, Eton et al. (1998) used the VMIQ and the VVIQ to measure the vividness of motor imagery and visual imagery among three athletic categories, including elite athletes, non-elite athletes, and non-athletes, and they found that VMIQ was able to differentiate these categories, while the VVIQ did not, indicating that no significant difference in visual imagery between the three group. In addition, the result that visual imagery scores were slightly higher in the non-dancers than the amateur dances, though the trend was not significant is consistent with studies reporting that individuals lower in motor imagery ability may use visual imagery more to enhance motor performance (Luzzeri, 2014).

### The individual differences between amateur dancers and non-dancers in the EVI and KIN

The results in relation to vividness of motor imagery revealed that amateur dancers scored significantly lower than non-dancers on EVI and KIN, suggesting that amateur dancers have higher ability to use visual motor and kinesthetic imageries. The reason for the superiority in abilities of EVI and KIN is that amateur dancers have more visual motor and kinesthetic imagery practice in dance movement learning and understand its benefits on dance performance. The results support the bioinformational theory proposed by Lang (1979). According to the theory, those who have greater experience of motor skills develop higher ability to produce the mental representation of movements (Barley, 2011). The present findings are compatible with recent studies using the high temporal resolution of event-related potentials (ERPs) to investigate the effects of expertise on dance movement representation (Orlandi et al., 2020a,b). Specifically, Orlandi et al. (2020a) revealed that professional dance practice may led to a refined kinesthetic motor representation of similar dance movements acquired during dance movement practice. Besides, the present findings are consistent with previous studies showing that athletes and professional musicians with higher levels of motor skills display higher ability of motor imagery (Cumming and Hall, 2002; Lotze et al., 2003; Gregg and Hall, 2006) and kinesthetic imagery (Féry and Morizot, 2000; Weinberg et al., 2003; Morris et al., 2005; Barley, 2011; Yu et al., 2016). The present study extends the literature by showing that amateur dancers who participate in dance activities just in a short time can display the superiority of their motor imagery ability in dance skill learning (Malouin et al., 2009). The results provide evidence for the notion that motor imagery ability is highly dynamic and modulated by imagery practice and levels of motor skills.

Amateur dancers were expected to better use of KIN than EVI. However, present results showed that amateur dancers equally used KIN and EVI. This unexpected result may be explained at least in part by dance movement characteristics, as performing a floor routine requires a reference frame integrating both motor visual and kinesthetic imageries to regulate posture and control body movement. These findings are in support of Guillot et al. (2004) who failed to found the significant differences between the use of EVI and KIN in gymnasts.

### The individual differences between amateur dancers and non-dancers in the dance movement reproduction task

The results showed that amateur dancers outperformed non-dancers on the dance movement reproduction task, which suggest a higher levels of motor imagery ability for amateur dancers than non-dancers. The inter-difference in dance performance may be ascribed in part to the different switching abilities between motor and visual imageries. In the present study, participants were required to learn the dance movements on cartoons, and then to reproduce the dance movements. To this end, participants may firstly use visual imagery to rehearse the gross form of body dance movements, and then switch to motor imagery from visual imagery to form the fine form of dance movements, such as the direction, speed, and gestures (Hardy and Callow, 1999; Barley, 2011). As this had already been shown in group of professional dancers (Golomer et al., 1999) and gymnasts (Guillot et al., 2004) who are thought to shift sensorimotor dominance from visual to motor imageries. Amateur dancers compared with non-dancers might have developed a higher switching ability from visual imagery to motor imagery during motor imagery practice in dance movement learning. This is congruent with the previous research which showed that participants switched backwards and forwards between visual imagery and motor imagery rather than use them concurrently (Collins et al., 1998; Olsson et al., 2008), since limited information are allowed to be displayed in working memory (Pylyshyn, 2003). However, studies provided evidence supporting that the experience of visual imagery and motor imagery can be concurrently (Cumming and Ste-Marie, 2001; Callow and Roberts, 2010; Floridou et al., 2022). Therefore, whether the individual differences in dance performance between amateur dancers and non-dancers is associated with the switching ability between visual imagery and motor imagery remain to be explored. It is worth noting that the present study has introduced a novel methodology for assessment of the motor imagery ability based on the video-analysis of target movements. This methodology could reduce the bias related to self-report measures.

### Limitations and contributions

Some limitations should consider addressing in the future study. First, the cross-sectional design in the study limited causal inferences. Thus, future studies could use multiple measures such as longitudinal design and intervention to determine the causality between individual differences in motor imagery ability and dance performance. Second, the conclusion was drawn only in a group of amateur dancers with relatively short 1.5 years dance experience, thereby, the generalization of the results to other dancer population is limited. The future study can explore the issue with dancers who have exposure to long-term dance training. Third, in the dance movement reproduction task we cannot determine the specific components of motor imagery. Hence, future research can use different motor imagery tasks (e.g., finger-tapping task using visual motor and kinesthetic imagery, respectively) to differentiate different components of motor imagery, i.e., the visual motor and kinesthetic imagery.

Despite limitations, this research has several theoretical and practical contributions. From a theoretical perspective, this research expands the knowledge on individual differences in motor imagery ability in non-athletes who vary in motor skill level, especially regarding visual and kinesthetic motor imageries, which might be implemented in motor imagery interventions and training programs. This study has the potential to advance our insights into why and which motor imagery ability contributes to the motor performance. This research also provides an empirical framework and approach to incorporate the objective measures of motor imagery into subjective measures of different aspects of motor imagery. From a practice perspective, our findings help us better understand the beneficial effect of motor imagery practice as an imagery technique on motor imagery during motor skill learning and sport performance. Given that motor imagery practice and motor imagery ability are significantly associated, more specialized and operationalized motor imagery practice should be developed, such as Neuromotor Task Training (NTT; Schmidt and Lee, 1999), motor teaching principles taxonomy (MTPT; Niemeijer et al., 2003), and PETTLEP-Based imagery interventions (Smith et al., 2007). For example, a recent study provided evidence that a 3-day training in dynamic neuro-cognitive imagery (DNI) greatly improved motor performance in college dance students (Abraham et al., 2019). Moreover, this research reveals that amateur athletes who have relatively high skill proficiency possess superior motor imagery ability, especially in visual and kinesthetic motor imageries. This indicates that the ability to imagine performing a movement improves as a function of acquired expertise with the motor skill, which could be treated as the basis of enhancing motor imagery ability.

## Data availability statement

The raw data supporting the conclusions of this article will be made available by the authors, without undue reservation.

## Ethics statement

The studies involving human participants were reviewed and approved by Ethics committee of Guangxi Minzu University. The patients/participants provided their written informed consent to participate in this study.

## Author contributions

XM, YX, and SH: conceptualization, software, and writing– original draft. XT: review and editing. MO: writing, supervision, and validation. All authors contributed to the article and approved the submitted version.

## Funding

This work was supported by the National Science Foundation of China (grant number: 61563003) to YX, the 2021 Research Project of Philosophy and Social Science Planning of Guangxi Zhuang Autonomous Region of China (grant number: 21FYY008), the Guangxi University Young and Middle-aged Teachers Scientific Research Basic Ability Improvement Project (grant number: 2021KY0143), and the Scientific Research Fund of Guangxi Minzu University (grant number: 2021MDSKYB03 and 2021SKQD31) to MO.

## Conflict of interest

The authors declare that the research was conducted in the absence of any commercial or financial relationships that could be construed as a potential conflict of interest.

## Publisher’s note

All claims expressed in this article are solely those of the authors and do not necessarily represent those of their affiliated organizations, or those of the publisher, the editors and the reviewers. Any product that may be evaluated in this article, or claim that may be made by its manufacturer, is not guaranteed or endorsed by the publisher.
